# Regulation Ratio: A Singular Multi-Omic Measurement of Gene Regulatory Mechanisms

**DOI:** 10.34133/csbj.0044

**Published:** 2026-04-22

**Authors:** Alexander Krohannon, Mansi Srivastava, Neel Sangani, Sarath Chandra Janga

**Affiliations:** ^1^ Department of Biomedical Engineering and Informatics, Luddy School of Informatics, Computing, and Engineering, Indiana University Indianapolis, Indianapolis, IN, USA.; ^2^ Department of Biology, College of Arts and Sciences, Indiana University, Bloomington, IN, USA.; ^3^ Department of Neurology, Indiana University School of Medicine, Indianapolis, IN, USA.; ^4^ Center for Computational Biology and Bioinformatics, Indiana University School of Medicine, Indianapolis, IN, USA.; ^5^ Department of Medical and Molecular Genetics, Indiana University School of Medicine, Indianapolis, IN, USA.

## Abstract

Gene expression is regulated through a complex interplay between transcriptional and post-transcriptional mechanisms, yet their relative contributions and relationships remain incompletely understood. In this study, we propose normalized metrics to quantify regulatory density at each of these 2 levels by integrating assay for transposase-accessible chromatin sequencing (ATAC-seq), RNA sequencing (RNA-seq), and protein occupancy profiling sequencing (POP-seq) data across 3 human cell lines—HEK293, HepG2, and K562—to assess gene-specific regulatory variations. This analysis revealed 3 distinct regulatory classes: predominantly post-transcriptionally regulated, predominantly transcriptionally regulated, and balanced genes. Using this metric, prominent associations between regulatory strategies and gene properties were uncovered, with transcriptionally regulated genes exhibiting greater length, post-transcriptionally regulated genes displaying higher isoform diversity and expression levels, and specific transcript types showing consistent enrichment patterns across regulatory categories. Remarkably, 55.8% of genes maintained identical regulatory classification across the 3 cell lines examined, with functional pathway analysis demonstrating high conservation of regulatory–functional relationships despite different cellular origins. This study provides a novel framework for understanding gene regulatory strategies and demonstrates that the relationship between gene properties and regulatory mechanisms represents a fundamental organizational principle that could transcend cell type specificity, with important implications for understanding dysregulation in disease states.

## Highlights

• Development of a singular metric for evaluating the propensity of gene-wise regulation at different levels of regulation.
• Generation of global RNA–protein occupancy profiles using POP-seq technique for 3 human cell lines.
• Majority of genes in cancer cells exhibited predominantly transcriptional regulation; in contrast, genes in noncancer cell line exhibited predominantly post-transcriptional regulation.

## Graphical Abstract

**Figure:**
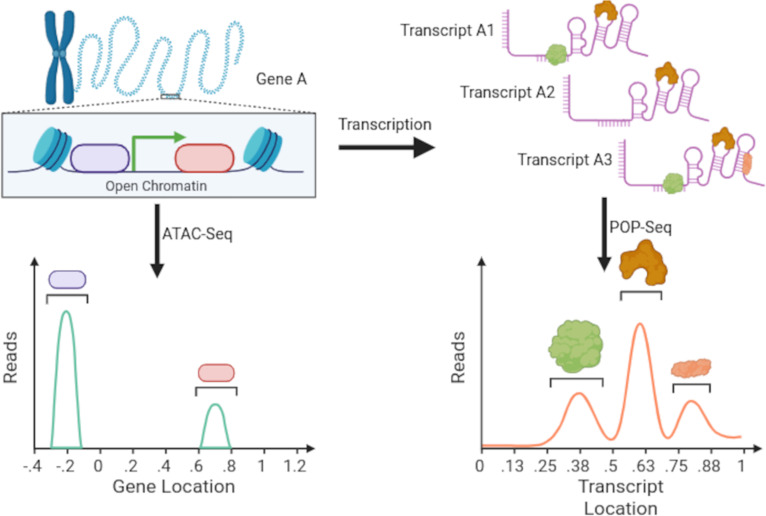

## Background

Gene expression in eukaryotic cells is governed by a complex network of regulatory mechanisms operating at multiple levels. These mechanisms collectively ensure proper spatial and temporal expression patterns of genes critical for cellular function, development, and response to environmental stimuli [1]. The eukaryotic regulatory landscape can be broadly categorized into transcriptional, post-transcriptional, translational, and post-translational control mechanisms, creating a sophisticated system that allows for precise orchestration of the cellular proteome [2].

Transcriptional regulation, the first level of control, involves mechanisms that determine whether and how efficiently a gene is transcribed into RNA [3]. This process begins with chromatin remodeling, where alterations in chromatin structure either facilitate or restrict the accessibility of DNA to transcriptional machinery [4]. Euchromatin, characterized by its open conformation, permits binding of transcription factors and RNA polymerase, while heterochromatin’s condensed structure impedes transcription [5]. Beyond accessibility, transcriptional regulation involves the coordinated action of transcription factors, enhancers, insulators, silencers, and promoters [6]. General transcription factors assemble at core promoters to form pre-initiation complexes, while sequence-specific transcription factors bind to proximal and distal regulatory elements, recruiting co-activators or co-repressors that modify local chromatin structure or directly interact with the transcriptional machinery [7]. This intricate network allows for combinatorial control of gene expression, where the presence or absence of specific regulatory proteins determines the rate of transcription initiation.

The advent of high-throughput sequencing technologies has revolutionized human understanding of these regulatory mechanisms by enabling genome-wide analysis of regulatory landscapes with unprecedented resolution [8]. Techniques such as chromatin immunoprecipitation sequencing (ChIP-seq) [9] and the more recent cleavage under targets and tagmentation (CUT&Tag) [10] have enabled direct mapping of histone modifications and DNA-binding proteins through antibody-mediated enrichment. Additionally, assay for transposase-accessible chromatin sequencing (ATAC-seq) has emerged as a powerful technique for mapping chromatin accessibility across the genome, requiring minimal sample input and providing insights into the locations of active regulatory elements such as promoters and enhancers [11]. ATAC-seq utilizes hyperactive Tn5 transposase to simultaneously fragment and tag accessible chromatin regions with sequencing adapters, creating a genome-wide profile of open chromatin that correlates strongly with transcriptional activity. This technique has proven invaluable for identifying cell type-specific regulatory elements and inferring transcription factor binding sites based on accessibility patterns and sequence motifs [12].

Post-transcriptional regulation occurs after RNA synthesis but prior to translation, encompassing processes that influence RNA processing, stability, and localization. Alternative splicing represents a critical post-transcriptional mechanism that generates multiple mRNA isoforms from a single gene, vastly expanding the proteome’s diversity beyond the genome’s coding capacity [13]. RNA-binding proteins (RBPs) play central roles by recognizing specific sequence or structural motifs within transcripts, regulating splicing decisions, polyadenylation site selection, and export from the nucleus [14]. Additionally, RBPs influence mRNA stability through interactions with the 5′ cap, poly(A) tail, and elements within the transcript body, particularly within 3′ untranslated regions (UTRs) [14]. Noncoding RNAs, especially microRNAs, provide another layer of control by targeting mRNAs through base-pairing interactions, typically resulting in translational repression or transcript degradation [15]. These post-transcriptional mechanisms collectively allow cells to rapidly adjust gene expression without initiating new transcription, providing flexibility and efficiency in responding to changing conditions.

Complementing ATAC-seq, protein occupancy profiling sequencing (POP-seq) provides a comprehensive view of RNA–protein interactions transcriptome-wide [16]. This technique involves isolation of protein-bound RNAs through phase separation, followed by ribonuclease digestion, proteinase and deoxyribonuclease digestion, and sequencing of the protected RNA fragments. POP-seq reveals binding sites of RBPs across all cellular transcripts, offering insights into post-transcriptional regulatory networks that influence RNA processing, localization, and stability. When combined with RNA sequencing (RNA-seq), which quantifies gene expression levels and identifies alternative splicing events, these technologies provide an integrated view of both transcriptional and post-transcriptional regulatory mechanisms operating across the genome [17].

**Fig. 1.**
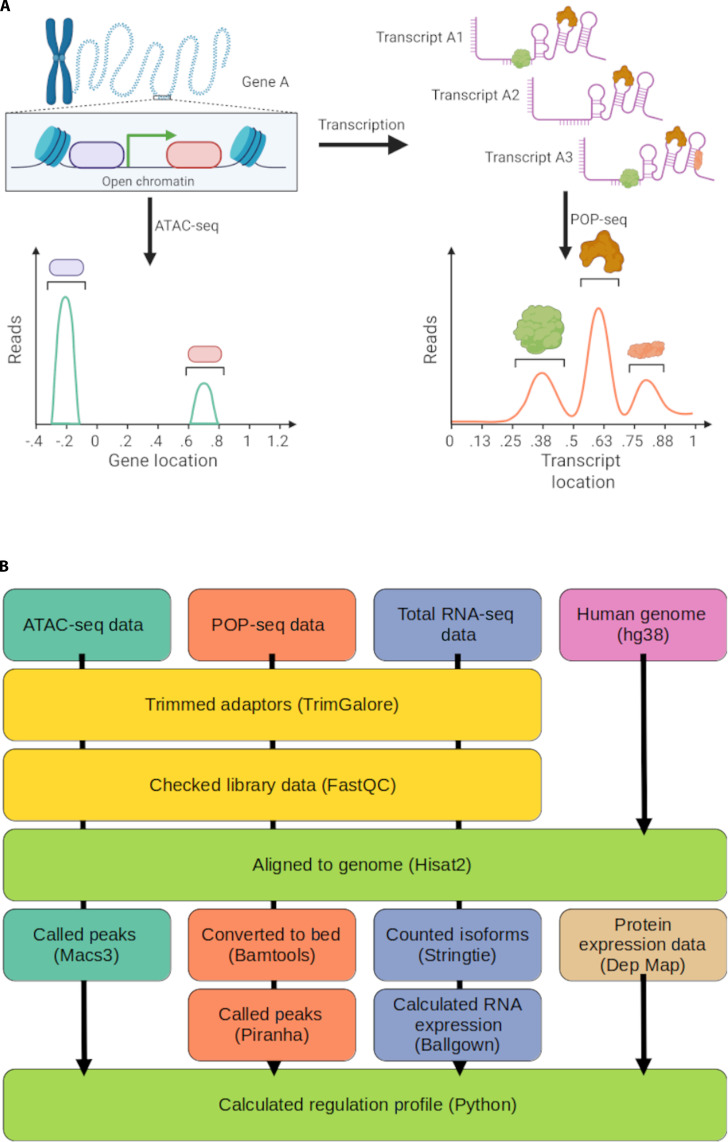
Experimental approach for gene regulatory profiling. (A) Schematic overview of complementary genomic approaches for identifying regulatory elements. Left panel: ATAC-seq (assay for transposase-accessible chromatin sequencing) detects regions of open chromatin in gene A. Right panel: POP-seq (protein occupancy profile sequencing) identifies RNA–protein interaction sites across multiple transcript isoforms of the same gene, with distinct binding profiles shown for different RNA-binding proteins. (B) Bioinformatics pipeline for integrated analysis of chromatin accessibility and RNA structural data.

The integration of these complementary sequencing approaches enables, for the first time, systematic comparison of regulatory patterns across different genes and cellular contexts (Fig. 1A). By correlating chromatin accessibility profiles from ATAC-seq with RNA–protein interaction maps from POP-seq, genes can be segregated based upon regulatory mode: genes predominantly regulated at the transcriptional level, those primarily controlled post-transcriptionally, and those with balanced regulation, subject to coordinated regulation at both levels. This integrated analysis revealed regulatory principles that govern gene expression, including relationships between gene architecture, function, and preferred regulatory mechanisms. Such comparative analyses across different cell types also illuminated how regulatory strategies are conserved or altered during development, differentiation, and disease progression.

## Materials and Methods

### Data acquisition

Publicly available ATAC-seq (SRR10319911 to SRR10319916) and total RNA-seq data (SRR10319891 to SRR10319896) were collected from SRA (PRJNA578731) for multiple cell lines, including human embryonic kidney 293 (HEK293), human liver cancer (HepG2), and human erythroleukemic (K562) cells [18,19]. In-house, protein occupancy profile sequencing (POP-seq) data were generated for these cell lines using the POP-seq technique as described previously [16]. POP-seq data for K562 cells comprised >60 million reads, which were generated previously by Srivastava et al. [16], while new POP-seq data corresponding to HEK293 and K562 cell lines were generated in this study (130 million and 75 million reads, respectively, in these cell lines) (Table 1). Protein expression data were obtained from the DepMap portal (https://depmap.org/portal), which provides comprehensive protein abundance measurements across multiple cell lines [20].

**Table 1.** Summary of next-generation sequencing files and their associated alignment metrics

Cell line	POP-seq 1 (SRA ID)	POP-seq 2 (SRA ID)	ATAC-seq 1 (SRA ID)	ATAC-seq 2 (SRA ID)	Total RNA-seq 1 (SRA ID)	Total RNA-seq 2 (SRA ID)
HEK293	133 million	153 million	54.1 million (SRR10319911)	54.1 million (SRR10319912)	24.6 million (SRR10319891)	25.5 million (SRR10319892)
‍Reads aligned (percentage)	128 million (95.7%)	148 million (96.6%)	52.0 million (96.2%)	52.7 million (97.4%)	24.2 million (98.3%)	25.1 million (98.3%)
‍HepG2	65.0 million (SRR10749115)	60.3 million (SRR10749116)	71.0 million (SRR100319913)	49.8 million (SRR100319914)	28.4 million (SRR10319893)	25.1 million (SRR10319894)
‍Reads aligned (percentage)	57.6 million (88.5%)	52.5 million (87.1%)	69.0 million (97.0%)	48.1 million (96.6%)	27.9 million (98.5%)	24.7 million (98.4%)
‍K562	81.5 million	75.6 million	58.4 million (SRR10319915)	83.6 million (SRR10319916)	24.5 million (SRR10319895)	25.1 million (SRR1039896)
‍Reads aligned (percentage)	77.5 million (95.1%)	71.9 million (95.1%)	56.7 million (97.0%)	80.8 million (96.6%)	24.1 million (98.1%)	24.7 million (98.2%)

### Cell culture

HEK293 and K562 cells were obtained from the American Type Culture Collection (ATCC). Cells were cultured in Dulbecco’s minimal essential medium (DMEM; Gibco) supplemented with 10% heat-inactivated fetal bovine serum (FBS; Atlanta Biologicals) along with 1% antibiotics (penicillin 5,000 U/ml, streptomycin 5,000 μg/ml). All cells were maintained at 37 °C and 5% CO_2_ in a humidified incubator, and fresh medium was replenished every alternate day until confluent.

### Quality control and preprocessing

Initial quality assessment of all sequencing data was performed using FastQC (v0.12.1) [21]. Reads with Phred quality scores below 33 were discarded from further analysis to ensure high-quality sequence data. RNA-seq samples were processed in paired-end mode to maintain read pair relationships. Adapter sequences were subsequently removed from all datasets using Trim Galore (v0.6.10) to eliminate potential technical artifacts [22,23].

### Sequence alignment and processing

All sequence data were aligned to the human reference genome (GRCh38/hg38) using HISAT2 (v2.2.0) [24,25]. For RNA-seq data, alignment was performed in paired-end mode to preserve mate pair information. The resulting sequence alignment map (SAM) files were converted to binary alignment map (BAM) format and sorted using SAMtools (v2.26.0) [26]. For POP-seq experiments, technical replicates were merged using SAMtools merge function to increase coverage depth and statistical power.

### ATAC-seq analysis

ATAC-seq peak calling was conducted by pooling both biological replicates within each cell line using MACS3 (3.0.0) [27]. The analysis was performed using parameters optimized for human genome size, with a shift size of 50 base pairs (bp) and an extension size of 100 bp. Peak coordinates and signal intensities were exported in bedGraph format for downstream analysis.

### POP-seq analysis

POP-seq peaks were detected on pooled both biological replicates using Piranha (v1.2.1) with the following parameters: no normalization, logarithmic covariates, and a bin size of 20 bp [28]. These parameters were selected to optimize detection of protein–RNA interaction sites while maintaining specificity.

### Transcriptome analysis

RNA-seq data were processed using 2 complementary approaches. Gene-level expression quantification was conducted in R using Ballgown (v2.4.2) to obtain transcript abundance estimates [29]. For isoform-level analysis, StringTie (v2.2.1) was utilized to assemble and quantify transcript isoforms, enabling the identification of alternative splicing events and isoform-specific expression patterns [30].

### Integrated regulatory analysis

A comprehensive regulatory profile was generated using a custom Python (v.3.9.21) script to integrate multiple data types (Fig. 1B). Human gene annotations were extracted from Ensembl BioMart and were integrated with POP-seq peaks, ATAC-seq accessibility data, and RNA-seq expression levels. Protein abundance data from DepMap were also incorporated [31].

### Quantitative metrics for studying preferential gene regulation

A density metric for transcriptional regulation was calculated by counting ATAC-seq significant peaks [false discovery rate (FDR)-corrected *P* < 0.05] within an expanded gene boundary (including 5,000 bp upstream and 500 bp downstream of each gene) and normalizing by gene length in kilobases (kb). This window was selected to as a compromise to capture proximal promoter binding, enhancers, and silencers, both upstream and downstream of the genes (see the Supplementary Materials). Peaks contained in overlapping expanded gene boundaries were counted for both genes. Post-transcriptional regulation was quantified by determining the density of significant (FDR-corrected *P* < 0.05) POP-seq peaks within gene boundaries, normalized by gene length in kb (Fig. 1A). Regulation ratio (RR) for each gene was calculated by dividing the post-transcriptional regulation metric by the transcriptional regulation metric described above [Eq. (1)].

$$\begin{array}{c} \mathrm{RR}=\frac{\frac{\#\text{ofSignificantATAC}-\text{seqpeaks}}{ge\text{nelength}}}{\frac{\#\text{ofSignificantPOP}-\text{seqpeaks}}{\text{genelength}}}=\frac{\#\text{ofSignificantPOP}-\text{seqpeaks}}{\#\text{ofSignificantATAC}-\text{seqpeaks}} \end{array}$$ (1)

### Statistical analysis and visualization

Statistical comparisons and analysis were performed with Python using a combination of argparse (1.1), os, numpy (v1.26.4), pandas (v1.2.5), scipy (1.13.1), and cliffs_delta (1.0.0) [32,33]. Data were visualized with Python using a combination of matplotlib (3.7.3), seaborn (0.11.2), and statannot (0.2.3) [34–36].

### Gene set enrichment

Pre-ranked gene set enrichment was performed on log_2_-transformed RR values using gene set enrichment analysis (GSEA) (v4.3.2) with the standard parameters [37].

## Results

### Regulation ratio

**Fig. 2.**
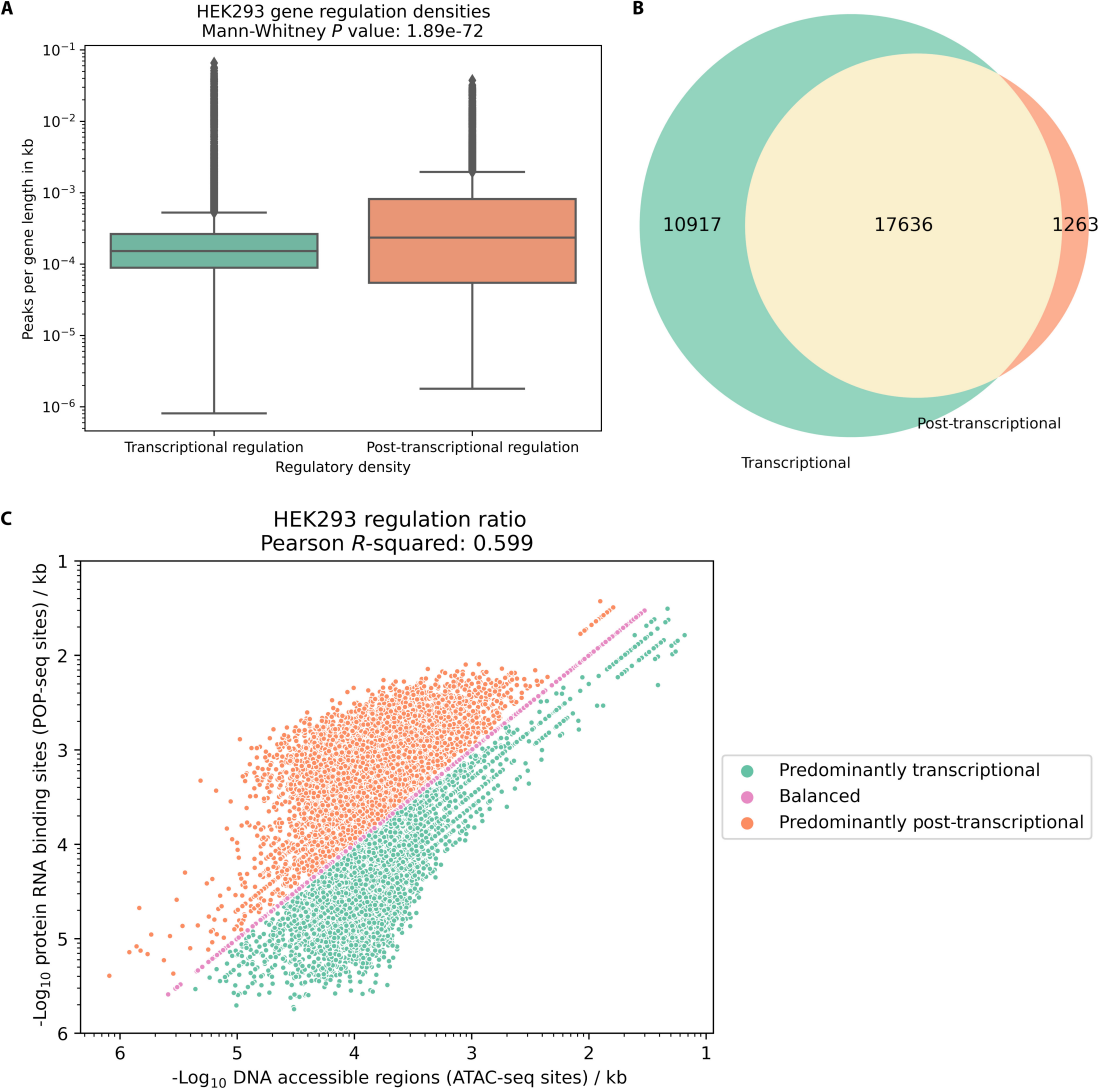
Comparison of transcriptional and post-transcriptional regulatory densities in HEK293. (A) Box plot showing the density of regulatory elements (peaks per gene length in kilobases) for genes regulated at transcriptional (green) and post-transcriptional (orange) levels. (B) Venn diagram illustrating the overlap between genes subject to transcriptional and post-transcriptional regulation. (C) Correlation analysis between DNA accessibility site (ATAC-seq) and protein–RNA binding site (POP-seq) densities, presented on log axes.

To enable direct comparisons between genes and regulatory mechanisms, singular metrics were required to quantitatively represent the degree of regulation at both transcriptional and post-transcriptional levels. The relative contributions of transcriptional and post-transcriptional regulation in HEK293, HepG2, and K562 cells were therefore quantified through normalized metrics developed for both regulatory mechanisms at individual gene level (see Materials and Methods and Table 1) and revealed significantly different distributions (Bonferroni-corrected Mann–Whitney *U* test, *P* = 1.89e−72, 5.53e−96, and 1.27e−128, respectively) in all 3 cell lines. Post-transcriptional regulation showed a greater than 4-fold (4.63) higher median density compared to transcriptional regulation, suggesting a predominant role for post-transcriptional control in HEK293 cell line (Fig. 2A). The other cell lines exhibited the opposite pattern of HEK293 with significantly higher median transcriptional regulation, with 2.52- and 2.58-fold change, possibly due to the embryonic (HEK293) versus developed stages (HepG2 and K562) of these specific cell lines 1.

While these individual metrics provided valuable insights into regulatory mechanisms, they did not fully capture the interplay between transcriptional and post-transcriptional control for any given gene. To develop a more comprehensive understanding of gene regulatory fate, the analysis focused on genes that exhibited both forms of regulation. Of the 29,800 genes analyzed in HEK293, 17,636 contained at least one ATAC-seq peak and at least one POP-seq peak, therefore were considered to pass the filter for subsequent RR analysis (Fig. 2B). The RR of each passing gene (14,648 in HepG2 and 12,929 in K562) was calculated and visualized by plotting the negative logarithm of post-transcriptional regulation metric against the transcriptional regulation metric (Figs. 2C and 4A and F). The analysis revealed not only a strong correlation between the 2 forms of regulation (Pearson *R*^2^: 0.599, 0.603, and 0.614, respectively) but also the existence of 3 distinct regulatory populations: predominantly post-transcriptionally regulated genes (RR > 1, 49.1%, 34.4%, and 31.5%, respectively), genes with regulatory balance (RR = 1, 9.87%, 8.23%, and 9.13%, respectively), and predominantly transcriptionally regulated genes (RR < 1, 41.0%, 57.3%, and 59.3%, respectively). To establish the biological significance of these regulatory classifications, fundamental gene characteristics were analyzed across different regulatory groups, namely, those that are predominantly transcriptionally regulated and predominantly post-transcriptionally regulated.

### Transcript type distribution of regulatory modes

**Fig. 3.**
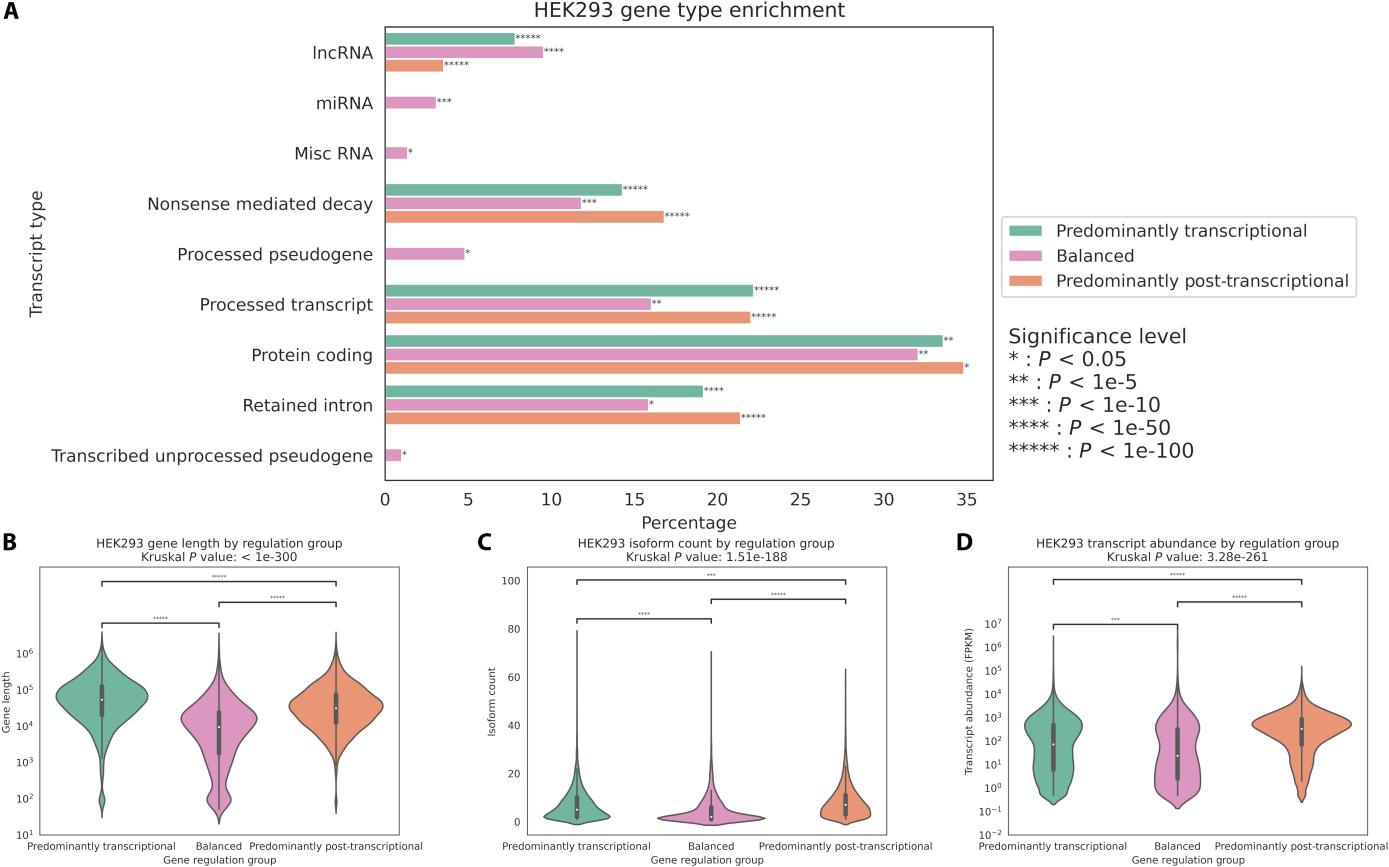
Distribution of transcript types across regulatory categories. (A) Percentage distribution of transcript types across predominantly transcriptional (green), balanced (pink), and predominantly post-transcriptional (orange) regulatory categories. (B) Violin plot comparing gene lengths (log_10_ scale) across the 3 regulatory categories. (C) Violin plot showing isoform count distributions across regulatory categories. (D) Violin plot showing the distribution of transcript abundance (in FPKM) across regulatory categories.

To characterize the distribution of transcript types across regulatory categories, Ensembl transcript annotations were mapped to genes within each regulatory group and quantified as percentages of total transcripts. After filtering for transcript types with greater than 1% abundance, enrichment analysis was performed using the Mann–Whitney *U* test, with human Ensembl transcript type annotations serving as the background distribution (Fig. 3A). This analysis revealed several significant patterns in the relationship between transcript type and regulatory mechanism. Protein-coding transcripts represented the largest fraction across all categories (greater than 30%) and were slightly underrepresented in each category, especially in genes with balanced regulation [*P* = 8.67e−6, odds ratio (OR) = 0.842]. Processed transcripts and retained intron transcripts both demonstrated high abundance in the predominantly post-transcriptionally regulated group (greater than 20%) and strong statistical enrichment in the predominantly post-transcriptionally regulated category (*P* = 2.41e−305, OR = 1.99 and *P* = 8.26e−204, OR = 1.75, respectively), suggesting a robust mechanistic link between post-transcriptional control and alternative RNA processing. Genes subject to nonsense-mediated decay (NMD) showed significant enrichment in both predominantly transcriptionally regulated and predominantly post-transcriptionally regulated categories (*P* = 7.15e−149, OR = 1.92 and *P* < 1e−300, OR = 2.33, respectively), suggesting complex regulatory control of these potentially deleterious transcripts. The balanced regulatory category uniquely contained all processed pseudogenes, miscellaneous RNAs, and miRNAs, each showing moderate but significant enrichment (*P* = 1.76e−2, OR = 1.23, *P* = 6.58e−3, OR = 1.56, and *P* = 6.01e−31, OR = 4.50, respectively), indicating that these noncoding RNA classes may require more balanced regulatory mechanisms.

Transcript type distribution and enrichment analyses in HepG2 and K562 cells revealed patterns consistent with those observed in HEK293 cells (Fig. 5A and B). Statistical evaluation of transcript type enrichment demonstrated significant association between predominantly post-transcriptionally regulated genes and NMD transcripts as well as retained intron transcripts (*P* < 1e−100, OR > 1.81) for all associations in both cell lines. Conversely, predominantly transcriptionally regulated genes showed significant enrichment for processed transcripts (*P* < 1e−300, OR > 2.08), while balanced genes exhibited significant associations with miRNAs (*P* < 1e−21, OR > 4.32).

### Gene length distribution of regulatory modes

**Fig. 4.**
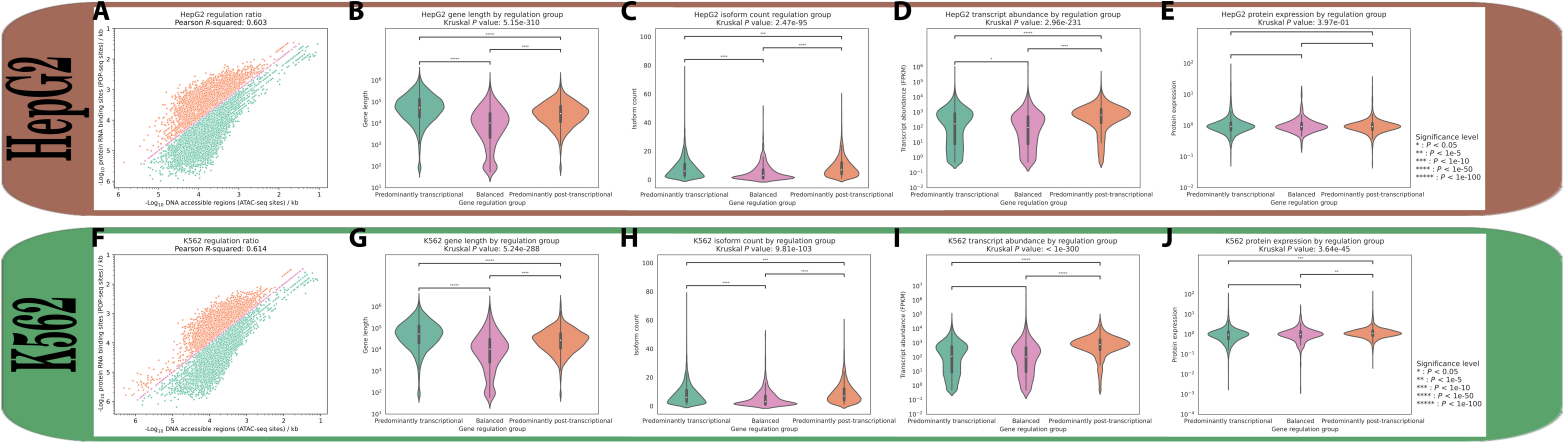
Comparative analysis of gene regulatory characteristics in HepG2 and K562 cell lines. (A and F) Correlation analysis between DNA accessibility (ATAC-seq) and protein–RNA binding (POP-seq) sites in HepG2 (A) and K562 (F) cell lines. (B and G) Violin plots comparing gene lengths (log_10_ scale) across regulatory categories in HepG2 (B) and K562 (G) cell lines. (C and H) Violin plots showing isoform count distributions across regulatory categories in HepG2 (C) and K562 (H) cell lines. (D and I) Violin plots of transcript abundance (log_10_ scale) across regulatory categories in HepG2 (D) and K562 (I) cell lines. (E and J) Violin plots showing protein expression levels (log_10_ scale) across regulatory categories in HepG2 (E) and K562 (J) cell lines.

Analysis of gene length across regulatory categories revealed distinct architectural features associated with different modes of gene regulation (Fig. 3B). Genes under predominant transcriptional control exhibited significantly higher lengths (median ~54,000 bp) compared to those with balanced regulation (median ~9,400 bp), while genes dominated by post-transcriptional regulation showed intermediate lengths (median ~31,000 bp) with greater length variability. A Kruskal–Wallis test demonstrated that gene lengths differed significantly across these regulatory categories (*P* < 1e−300). Subsequent pairwise comparisons using Mann–Whitney *U* tests confirmed significant differences between all pairs of regulatory groups [*P* < 1e−100 for all comparisons, with Cliff’s delta values (CD) of 0.595, 0.469, and 0.206 for predominantly transcriptionally controlled versus balanced, predominantly post-transcriptionally controlled versus balanced, and predominantly transcriptionally controlled versus predominantly post-transcriptionally controlled, respectively), indicating distinct length distributions for each regulatory strategy. This same pattern was also observed in HepG2 (medians: transcriptional: 54,000 bp, balanced: 10,000 bp, and post-transcriptional: 29,000 bp) and K562 (medians: transcriptional: 54,000 bp, balanced: 10,000 bp, and post-transcriptional: 27,000 bp) cell lines (Mann–Whitney *U* test, *P* < 1e−50 for all pairwise comparisons, CD: 0.555, 0.387, and 0.264 for HepG2, and 0.530, 0.349, and 0.288 for K562, respectively) (Fig. 4B and G).

**Fig. 5.**
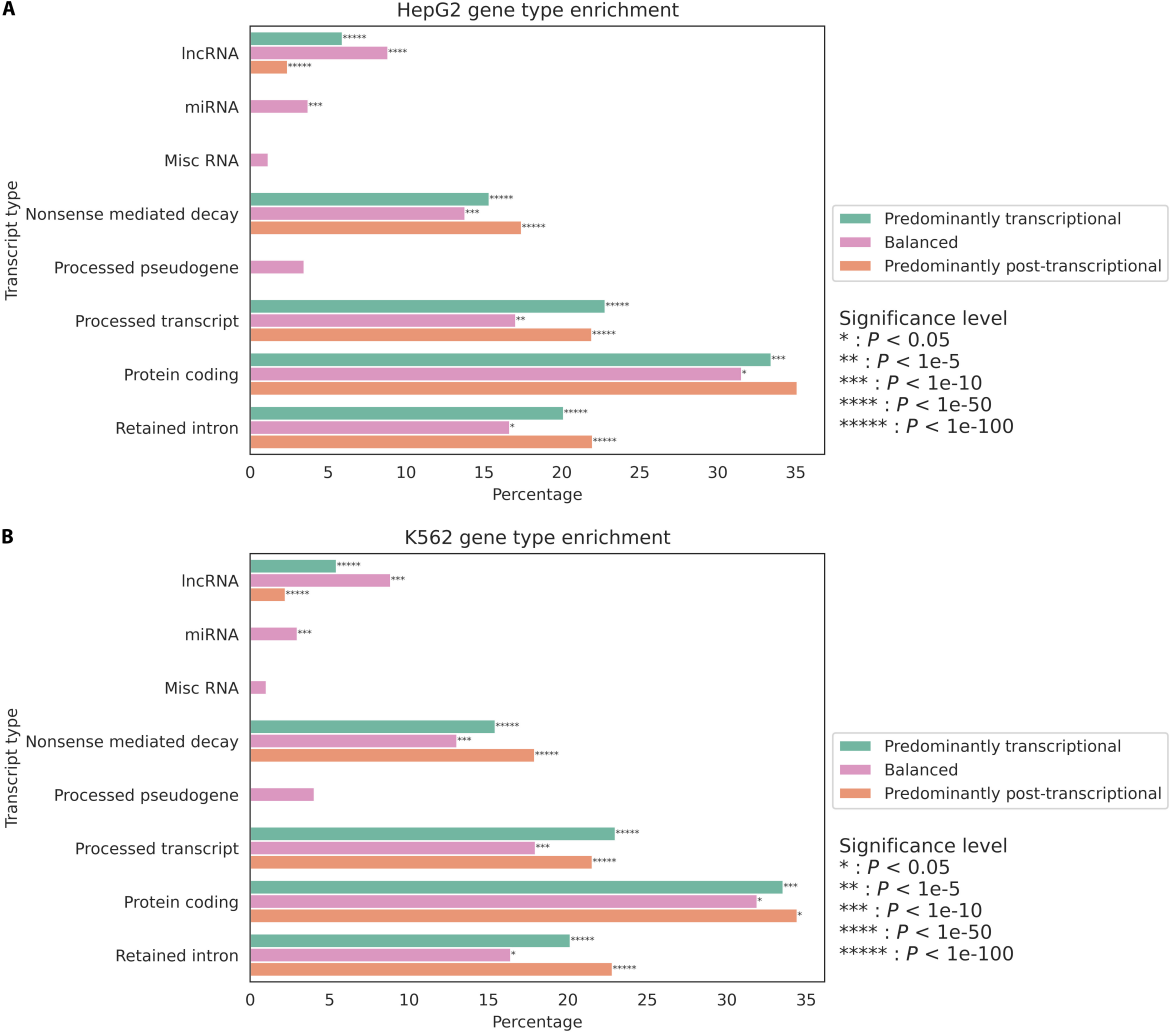
Comparative analysis of transcript type distribution across regulatory categories in HepG2 and K562 cell lines. (A) Percentage distribution of transcript types across regulatory categories in HepG2 cells. (B) Percentage distribution of transcript types across regulatory categories in K562 cells.

### Isoform distribution of regulatory modes

Further investigation of genetic complexity across regulatory categories revealed significant differences in transcript isoform counts (Fig. 3C). Post-transcriptionally regulated genes displayed the highest median number of isoforms, followed by transcriptionally regulated genes, while balanced genes showed the lowest isoform diversity (7, 5, and 2, respectively). A Kruskal–Wallis test confirmed significant differences in isoform distributions across regulatory categories (*P* = 1.51e−188). Pairwise Mann–Whitney *U* tests demonstrated that each regulatory group maintained distinct isoform distributions (*P* < 1e−10 for all comparisons, CD: 0.319, 0.429, and −0.130 for predominantly transcriptionally controlled versus balanced, predominantly post-transcriptionally controlled versus balanced, and predominantly transcriptionally controlled versus predominantly post-transcriptionally controlled, respectively). The elevated isoform count in post-transcriptionally regulated genes aligns with the expected role of post-transcriptional processes in generating transcript diversity through mechanisms such as alternative splicing. This same pattern was also observed in HepG2 (medians: transcriptional: 6, balanced: 3, and post-transcriptional: 7) and K562 (medians: transcriptional: 6, balanced: 3, and post-transcriptional: 7) cell lines (Mann–Whitney *U* test, *P* < 1e−10 for all pairwise comparisons, CD: 0.313, 0.381, and −0.076 for HepG2, and 0.309, 0.404, and −0.107 for K562, respectively) (Fig. 4C and H).

### Transcript abundance distribution of regulatory modes

Examination of transcript abundance across regulatory categories revealed striking differences in gene expression levels [fragments per kilobase of transcript per million mapped reads (FPKM)] (Fig. 3D). Predominantly post-transcriptionally regulated genes exhibited markedly higher median transcript abundance compared to both transcriptionally regulated and balanced genes (310, 34, and 6.75, respectively). Statistical analysis using the Kruskal–Wallis test confirmed highly significant differences in expression distributions across regulatory categories (*P* = 3.28e−261). Pairwise Mann–Whitney *U* tests demonstrated that each regulatory group maintained distinct expression profiles (*P* < 1e−10 for all comparisons, CD: 0.179, 0.488, and −0.336 for predominantly transcriptionally controlled versus balanced, predominantly post-transcriptionally controlled versus balanced, and predominantly transcriptionally controlled versus predominantly post-transcriptionally controlled, respectively). The substantially elevated expression of predominantly post-transcriptionally regulated genes suggests that these genes not only display greater isoform diversity but also maintain higher overall transcript levels, potentially reflecting their biological importance and the complex interplay between regulatory mechanisms and gene expression output. This same pattern was also observed in HepG2 (medians: transcriptional: 173, balanced: 97, and post-transcriptional: 651) and K562 (medians: transcriptional: 116, balanced 106, and post-transcriptional: 783) cell lines (Mann–Whitney *U* test, *P* < 1e−5 for all pairwise comparisons, CDs: 0.147, 0.513, and −0.369 for HepG2, and 0.09, 0.567, and −0.511 for K562, respectively) (Fig. 4D and I).

### Protein expression distribution of regulatory modes

The relationship between regulatory mechanisms and protein expression was also investigated using protein expression data obtained from the DepMap portal. HEK293 cells were excluded from this analysis due to the absence of corresponding data in the DepMap database. Analysis of protein expression across regulatory categories in HepG2 cells revealed no significant differences (Kruskal–Wallis test, *P* = 0.397, CD: 0.998). In contrast, K562 exhibited statistically significant differences in protein levels across regulatory groups (Kruskal–Wallis test, *P* = 3.64e−45, CD: 0.998). Subsequent pairwise comparisons using Mann–Whitney *U* tests confirmed that predominantly post-transcriptionally regulated genes in K562 cells produced marginally higher protein levels compared to both balanced genes (1.14-fold increase, *P* = 4.00e−7, CD: 1.00) and predominantly transcriptionally regulated genes (1.20-fold increase, *P* = 2.81e−45, CD: 1.00). Although the data for protein expression have been limited to only 2 cell lines in this analysis, this observation suggests that genes that are predominantly post-transcriptionally regulated are more likely to exhibit higher protein pools.

### Comparative functional analysis of regulatory modes

**Fig. 6.**
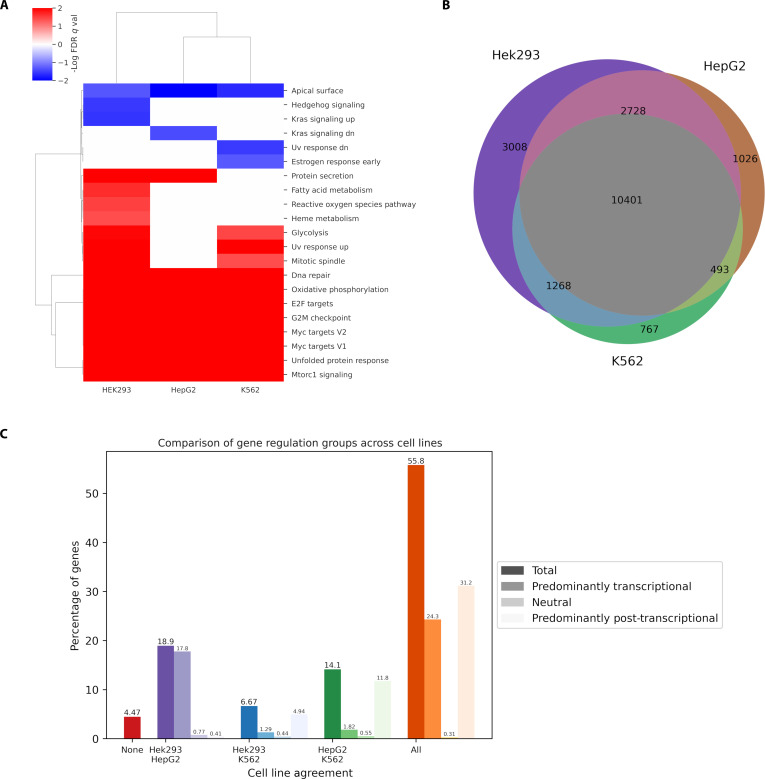
Cross-cell line analysis of gene regulatory patterns and pathway enrichment. (A) Hierarchical clustering heatmap showing log_10_ FDR-corrected *P* values across HepG2, K562, and HEK293 cell lines. (B) Three-way Venn diagram illustrating the overlap of regulated genes across HEK293, HepG2, and K562 cell lines. (C) Bar chart comparing the percentage of genes in different regulatory categories based on cell line agreement.

To investigate the functional implications of differential regulatory mechanisms across cell types, pre-ranked GSEA was performed using log_2_-transformed RR values from all 3 cell lines. Analysis of significantly enriched gene sets (FDR *q* < 0.05) revealed remarkable consistency across HEK293, HepG2, and K562 cells (Fig. 6A). Among the 21 gene sets showing significant enrichment in at least one cell line, 9 (42.9%) were consistently enriched across all 3 cell types, with 8 positively enriched (RR > 1) and 1 negatively enriched (RR < 1). Cell type-specific enrichment was similarly distributed, with 8 gene sets (38.1%) uniquely enriched in a single cell line. Furthermore, the directionality of enrichment demonstrated near-perfect concordance, with every gene set (13, 61.9%) maintaining identical enrichment patterns across all cell lines in which they were observed.

To quantify the conservation of regulatory mechanisms across cell types, comparative analysis was performed on the regulatory patterns of genes common to all 3 cell lines with RR serving as a filer. From the initial gene set (17,405 in HEK293, 14,648 in HepG2, and 12,929 in K562), 10,401 genes were identified as present across all 3 cell lines, enabling direct cross-comparison of regulatory strategies (Fig. 6B). Agreement analysis of these shared genes revealed substantial conservation, with 55.8% demonstrating identical regulatory classification across all 3 cell lines (Fig. 6C). Among genes with partial agreement, the strongest concordance was observed between HEK293 and HepG2 cells (18.9%), followed by HepG2 and K562 cancer cell lines (14.1%), and HEK293 and K562 agreement represented 6.67% of the common gene set. Notably, only 4.47% of genes exhibited completely discordant regulatory patterns with no agreement between any cell lines.

## Discussion

Utilizing bulk paired ATAC-seq and total RNA-seq with POP-seq, the interplay between transcriptional and post-transcriptional regulatory mechanisms was explored. To that end, normalized metrics for quantifying regulatory density, as well as a unified metric for evaluating gene-specific regulation were developed. By developing these metrics, this study demonstrates not only the existence of distinct regulatory modes but also their relationship to gene architecture, expression profiles, and functional characteristics.

### Regulatory landscape and gene classification

This framework was applied to cell line data, resulting in the categorization into 3 distinct regulatory classes: predominantly post-transcriptionally regulated, predominantly transcriptionally regulated, and balanced. This classification system provides a novel framework for understanding gene regulatory strategies. The strong correlation observed between transcriptional and post-transcriptional regulation suggests coordinated interaction between these mechanisms, rather than independent operation. The observed predominance of post-transcriptional regulation in HEK293 cells contrasts with the pattern observed in HepG2 and K562 cells. This difference may reflect the noncancerous origin of HEK293 cells compared to the cancer-derived HepG2 and K562 lines, suggesting potential dysregulation of post-transcriptional mechanisms in cancer cells.

### Gene architecture and regulation strategy

Predominantly transcriptionally regulated genes exhibited substantially longer sequences compared to balanced genes and post-transcriptionally regulated genes, regardless of cell line. This pattern suggests that gene length may influence regulatory strategy. A possible explanation for this trend could be that since longer genes contribute to higher investments of cellular resources such as raw materials, energy, and time, they are likely to be regulated at multiple levels prior to their production of functional products. The consistency of this relationship across all 3 studied cell lines, despite their distinct origins and phenotypic characteristics, strongly suggests that this pattern represents a fundamental biological constraint rather than a cell type-specific adaptation.

Analysis of transcript isoform counts revealed that predominantly post-transcriptionally regulated genes display the highest isoform diversity, followed by transcriptionally regulated genes, with balanced genes showing the lowest diversity. This pattern aligns with the established role of post-transcriptional processes in generating transcript diversity through mechanisms such as alternative splicing, polyadenylation, and RNA editing [38–40]. The higher isoform diversity in transcriptionally regulated genes compared to balanced genes may reflect the relationship between complex promoter architecture and alternative transcription start site selection [41,42].

### Expression levels and regulatory categories

A particularly striking finding was the substantially higher expression levels observed in predominantly post-transcriptionally regulated genes compared to other regulatory categories. This dramatic difference suggests that post-transcriptional regulation may be particularly important for highly expressed genes, potentially providing finer control over final expression levels and enabling rapid responses to changing cellular conditions without requiring transcriptional reinitiation [43,44].

The analysis of protein expression across regulatory categories revealed cell type-specific patterns. While no significant differences were observed in HepG2 cells, K562 cells demonstrated higher protein levels for predominantly post-transcriptionally regulated genes compared to other categories. This finding highlights the complex relationship between regulatory mechanisms and protein output, suggesting that the impact of regulatory strategy on final protein abundance may depend on cell type-specific factors. The modest fold increase in protein levels despite the substantially higher transcript abundance observed in post-transcriptionally regulated genes suggests extensive buffering of transcript levels during translation, consistent with previous studies showing imperfect correlation between mRNA and protein abundance [45,46].

### Transcript types and regulatory categories

The distribution of transcript types across regulatory classifications provides further insight into the functional significance of different regulatory strategies. Protein-coding transcripts, while abundant across all categories, showed significant enrichment in predominantly transcriptionally regulated genes. This suggests that precise transcriptional control may be particularly important for protein-coding genes, potentially ensuring appropriate temporal and spatial expression patterns.

The significant enrichment of processed transcripts, retained intron transcripts, and NMD transcripts in predominantly post-transcriptionally regulated genes highlights the central role of post-transcriptional mechanisms in RNA processing and quality control. This pattern suggests that genes producing these transcript types rely heavily on RNA-binding protein interactions to regulate splicing decisions, intron retention, and surveillance of potentially deleterious transcripts [47,48]. The consistent enrichment of these transcript types across both noncancerous and cancerous cell lines demonstrates the robustness of our proposed metrics and their ability to identify biologically relevant patterns.

The predominance of transcriptional control for long noncoding RNAs (lncRNAs) may reflect their often-precise spatiotemporal expression patterns and cell type specificity, which are critical for their regulatory functions. It is noteworthy to mention that a significant fraction of the lncRNAs have significantly fewer exons, thereby not necessitating much post-transcriptional control [49]. Transcriptional control, through mechanisms such as promoter architecture, enhancer interactions, and chromatin modifications, may provide the necessary precision for coordinating lncRNA expression with their target genes [50,51]. The conservation of this pattern across both noncancerous and cancerous cell lines suggests a fundamental regulatory principle that transcends malignant transformation. However, the implications of this regulatory pattern in cancer contexts warrant deeper consideration. Cancer cells frequently exhibit dysregulation of lncRNAs, with many lncRNAs serving as oncogenes or tumor suppressors [52,53]. This finding in cancer cells indicates that their dysregulation of lncRNAs in malignancy may originate primarily from alterations in transcription factor networks, chromatin structure, or epigenetic modifications rather than from changes in post-transcriptional processing [51].

The exclusive presence of processed pseudogenes, miscellaneous RNAs, and miRNAs in the balanced regulatory category across all 3 cell lines represents an intriguing pattern with significant implications for noncoding RNA regulation. This consistent distribution suggests that these noncoding RNA classes require balanced input from both transcriptional and post-transcriptional mechanisms, rather than predominantly relying on either regulatory mode. Such balanced regulation may reflect the diverse functional roles of these transcripts in gene expression networks, where coordinated control at multiple levels ensures appropriate expression patterns [54].

For miRNAs in particular, this balanced regulation is consistent with their biogenesis pathway, which involves both transcriptional regulation of primary miRNA transcripts and extensive post-transcriptional processing by Drosha and Dicer [55]. The conserved balanced regulatory pattern for processed pseudogenes and transcribed unprocessed pseudogenes may indicate evolved regulatory mechanisms that maintain appropriate expression levels of these potentially regulatory noncoding RNAs, preventing deleterious effects that could arise from their overexpression. The conservation of this pattern across cell lines with different phenotypic characteristics and origins (including both cancerous and noncancerous cells) further underscores the fundamental nature of this regulatory strategy for these transcript classes.

### Conservation of regulatory mechanisms across cell lines

Perhaps the most remarkable finding of this study is the high degree of conservation in regulatory patterns across distinct cell lines. Despite the different origins and phenotypic characteristics of HEK293, HepG2, and K562 cells, 55.8% of genes maintained identical regulatory classification across all 3 cell lines. This conservation extends to the functional implications of regulatory mechanisms, with 38.1% of significantly enriched gene sets showing consistent enrichment across all 3 cell types. The strong concordance in regulatory patterns suggests that the relationship between gene properties and regulatory mechanisms may represent an organizational principle of gene expression control in immortal cell lines.

The consistent positive GSEA enrichment of 8 key pathways across HEK293, HepG2, and K562 cells provides valuable insights into regulatory conservation in cancerous and stem-like cellular contexts. The enrichment of proliferation-associated pathways including “Myc Targets V1/V2” [normalized enrichment score (NES): {2.57, 2.45}, {3.03, 3.11}, and {2.85, 2.80} for HEK293, HepG2, and K562 for Myc Targets V1 and V2, respectively] [56–60], “E2F Targets” (NES: 2.41, 3.04, and 2.50) [57,61–65], “G2M Checkpoint” (NES: 2.41, 2.99, and 2.76), and “mTORC1 Signaling” (NES: 1.91, 2.26, and 2.17) across these distinct cell types reflects the heightened proliferative capacity common to both cancer cells (HepG2 and K562) and embryonic-derived cells with stem-like properties (HEK293). This suggests that genes controlling cell cycle progression maintain specific post-transcriptional regulatory architectures regardless of the specific oncogenic drivers or tissue origins [66].

Similarly, stress response pathways including “DNA Repair” (NES: 2.05, 2.28, and 1.89 for HEK293, HepG2, and K562, respectively) and “Unfolded Protein Response” (NES: 2.00, 2.31, and 2.34) showing consistent enrichment may indicate conserved regulatory mechanisms for managing increased genomic instability and proteotoxic stress common in these cellular contexts [67–72]. The preservation of regulatory strategies for these pathways across different cancer-derived and stem-like cells suggests fundamental principles governing gene regulation in contexts of heightened proliferation and metabolic activity, rather than tissue-specific regulatory programs.

In stark contrast to Conversely, pathways related to cellular identity and developmental processes (Apical Surface, Hedgehog Signaling, Epithelial Mesenchymal Transition, Myogenesis, Notch Signaling), cell–environment interactions (Angiogenesis, Complement, Coagulation), and certain signaling pathways (KRAS Signaling Up/Down, Estrogen Response Early) consistently showed predominant transcriptional regulation (RR < 1). This pattern indicates that processes requiring stable, long-term expression programs or tissue-specific control may benefit from precise transcriptional regulation. The remarkable conservation of these regulatory orientations across diverse cell types suggests that the relationship between regulatory strategy and functional category represents a fundamental organizational principle of gene expression control, rather than a cell type-specific adaptation. This consistency further suggests that the evolution of regulatory mechanisms has been shaped by the functional requirements of the pathways they control.

GSEA also provides valuable insights into the functional implications of different regulatory modes. The high consistency in enrichment patterns across cell lines reinforces the notion that regulatory strategies are intrinsically linked to gene function rather than being determined solely by cellular context. The observation that only a single gene set (Androgen Response) showed unique enrichment in one cell line further emphasizes the conservation of regulatory–functional relationships [73,74].

### Limitations and future directions

While this study provides valuable insights into gene regulatory strategies, several limitations should be acknowledged. First, this analysis focused on steady-state measurements of chromatin accessibility and RNA-binding protein occupancy, which do not fully capture the dynamic nature of gene regulation. Future studies incorporating time-course and single-cell measurements following cellular perturbations would enhance our understanding of regulatory dynamics.

Second, the metrics for transcriptional and post-transcriptional regulation, while informative, represent simplified approximations of complex processes. Furthermore, this metric is unable to take either the directionality or the magnitude of any interaction into account. The integration of additional data types, including ChIP-seq for histone modifications, RNA modification profiling, and ribosome profiling, could provide insight into particular interactions; however, systemic interrogations would still be required for determination of magnitude and indeed directionality of some interactions.

Finally, while this study included 3 different cell lines, all were derived from human tissues and maintained in culture. Investigation of regulatory patterns in primary tissues and across different species would help determine the universality of our findings. Future studies employing single-cell sequencing techniques could provide higher-resolution insights into cell-specific regulatory mechanisms that may be masked in bulk analyses. This approach would be particularly powerful when combined with spatial transcriptomics, allowing for the examination of regulatory dynamics within tissue microenvironments and revealing how cellular interactions influence regulatory strategies. Additionally, applying our analytical framework to stem cell differentiation models could elucidate how regulatory mechanisms evolve during lineage commitment and cellular specialization, potentially identifying critical regulatory transitions governing cell fate decisions. Such investigations could reveal whether the regulatory classifications we observed represent stable cellular properties or dynamic states that shift during development and differentiation.

## Conclusion

This comprehensive analysis of transcriptional and post-transcriptional regulatory mechanisms across HEK293, HepG2, and K562 cell lines reveals a striking conservation of regulatory patterns despite their distinct cellular origins. By developing novel metrics to quantify regulatory density and classify genes into predominant regulatory categories, fundamental relationships were identified between gene architecture, expression profiles, and regulatory strategies that transcend cell type specificity. The robust correlation between these regulatory modes suggests coordinated rather than independent operation, with specific gene properties—including length, isoform diversity, and transcript type—strongly associated with particular regulatory strategies. The remarkable conservation of regulatory–functional relationships across both noncancerous and cancer-derived cell lines, evidenced by consistent enrichment patterns in functional pathways, suggests that these regulatory principles represent fundamental organizational features of mammalian gene expression. Future investigations incorporating time-course measurements, additional data types, and primary tissue samples will further elucidate the dynamic interplay between transcriptional and post-transcriptional mechanisms in different cellular contexts, potentially revealing new insights into dysregulation in disease states.

## Data Availability

Raw and POP-seq data presented in this paper are accessible at Sequence Read Archive (SRA) ID: 16057836. Code used in this project is available from the following GitHub link:  https://github.com/Janga-Lab/Regulation_Ratio.
