# Supplementary material for: Regulation Ratio: A Singular Multi-Omic Measurement of Gene Regulatory Mechanisms
Source: Comput Struct Biotechnol J. 2026 Apr 22;35(1):0044. doi: 10.34133/csbj.0044 (PMC13100350; doi:10.34133/csbj.0044)
Supplement: Supplementary 1 — Supplementary Text Fig. S1 Tables S1 and S2 [file csbj.0044.f1.zip › Supplemental_Material.docx]

**Supplemental Material**

**Sensitivity Analysis of ATAC-seq Peak Window Selection**

To evaluate the robustness of the Regulation Ratio (RR) classification to the choice of ATAC-seq peak window, the analysis was repeated across five window configurations extending beyond the annotated transcription boundaries: 0 bp upstream / 0 bp downstream, 1,000 bp / 100 bp, 2,500 bp / 250 bp, 5,000 bp / 500 bp (selected for the primary analysis), and 10,000 bp / 1,000 bp. For each window, genes were classified into the three regulatory categories — Predominantly Transcriptionally Regulated, Balanced, and Predominantly Post-Transcriptionally Regulated — across all three cell lines (HEK293, HepG2, and K562), and the resulting category membership proportions are reported in Supplemental Table 1.

Both the Predominantly Transcriptionally Regulated and Predominantly Post-Transcriptionally Regulated categories demonstrated considerable robustness to variation in this parameter. Across all cell lines and window configurations, deviations from the 5,000 bp / 500 bp reference window were modest, with a maximum observed difference of 4.15% (Predominantly Transcriptionally Regulated, K562, 0 bp window) and an overall mean absolute difference of 1.62% (cell line-wise mean differences of 1.35%, 1.73%, and 1.79% for HEK293, HepG2, and K562, respectively). The Balanced category was particularly stable, with a group-wise mean absolute difference of only 0.27%, compared to 2.42% and 2.19% for the Predominantly Transcriptionally Regulated and Predominantly Post-Transcriptionally Regulated groups, respectively.

Notably, the direction of change across window sizes was consistent and biologically interpretable: expanding the ATAC-seq window progressively increased the proportion of genes classified as Predominantly Transcriptionally Regulated, as additional peaks from distal regulatory elements were captured within the broader window. The 5,000 bp / 500 bp window was therefore selected as a practical compromise that captures proximal promoter elements, nearby enhancers, and silencers while limiting the inclusion of regulatory elements more likely associated with neighboring genes.

**Gene-Level Agreement Analysis**

To further evaluate the robustness of the RR classification framework, a confusion matrix was constructed for each window configuration using the 5,000 bp / 500 bp window as the reference, and recall was calculated for each regulatory category across all three cell lines (Supplemental Table 2). This gene-level analysis revealed two distinct patterns of stability depending on regulatory category.

The Predominantly Transcriptionally Regulated and Predominantly Post-Transcriptionally Regulated categories again demonstrated strong robustness, with recall values deviating from the reference by no more than 16.2% across any window tested (K562 Predominantly Transcriptionally Regulated and HEK293 Predominantly Post-Transcriptionally Regulated, both at 0 bp). Group-wise mean absolute differences in recall were 5.58% and 5.80% for the Predominantly Transcriptionally Regulated and Predominantly Post-Transcriptionally Regulated categories, respectively, and cell-wise mean differences were 13.8%, 14.5%, and 14.6% for HEK293, HepG2, and K562. The overall mean absolute difference in recall across all groups and cell lines was 14.3%.

In contrast, the Balanced category exhibited substantially greater variability, with a group-wise mean absolute recall difference of 31.6% and individual deviations reaching as high as 66.9% (K562, 0 bp window). This heightened sensitivity is attributable to two compounding phenomena. First, modifying the ATAC-seq window directly alters the number of peaks captured per gene — narrowing the window reduces peak counts while expanding it increases them — thereby shifting the relative balance between transcriptional and post-transcriptional regulatory densities and displacing genes near the RR = 1 boundary. Second, altering the window also changes the number of genes that satisfy the inclusion criterion of possessing at least one ATAC-seq peak and at least one POP-seq peak. Consequently, a gene classified as Predominantly Post-Transcriptionally Regulated at the reference window may fall below the inclusion threshold entirely at a narrower window, and conversely, genes absent from the analysis at smaller windows may enter the gene set and be classified differently at larger ones. Because the Balanced category is defined by precise equality between transcriptional and post-transcriptional regulatory densities, it is inherently more sensitive to both of these effects than the two predominant categories.

**Gene Length Correlation Analysis**

To assess whether the Regulation Ratio could be confounded by a residual correlation between gene length and the number of observed peaks, a gene-wise Spearman correlation analysis was performed between gene length and peak count for both ATAC-seq (Supplemental Fig 1 (A, D, and G) and POP-seq (Supplemental Figure 1(B, E, and H) data across all three cell lines.

The two assays yielded strikingly divergent results. The number of observed ATAC-seq peaks showed a moderate positive correlation with gene length (R² = 0.592, 0.577, and 0.542, all p < 1E-300, for HEK293, HepG2, and K562, respectively), consistent with the rationale for length-normalization described in the Methods. In stark contrast, the number of observed POP-seq peaks showed little to no correlation with gene length (R² = 0.082, 0.043, and 0.016, with corresponding p-values of < 1E-300, < 1E-300, and 2.85E-112, for HEK293, HepG2, and K562, respectively). These observations suggest that while length-normalization was appropriate and necessary for the ATAC-seq metric, however, the POP-seq peak count appears to be largely independent of gene length, even prior to normalization.

**Correlation Between POP-seq Peak Count and Transcript Abundance**

To further investigate the decoupling of POP-seq peak counts from gene length, an additional gene-wise Spearman correlation analysis was performed between the number of observed POP-seq peaks and transcript abundance (FPKM) across all three cell lines (Supplemental Fig 1 (C, F, and I). Despite the intuitive expectation that more abundant transcripts would present proportionally more opportunities for protein-RNA interactions and thus yield greater numbers of detected peaks, the results revealed little to no correlation across all three cell lines (R² = 0.135, 0.151, and 0.246, all p < 1E-300, for HEK293, HepG2, and K562, respectively).

**Discussion:**

The three correlation analyses described above ATAC-seq peaks versus gene length (moderate, R² ~0.57–0.59), POP-seq peaks versus gene length (weak, R² ~0.02–0.08), and POP-seq peaks versus transcript abundance (weak, R² ~0.14–0.25) together paint a coherent and interpretable picture when considered in the context of the fundamental biology of each assay and the specific characteristics of immortalized cell lines.

The moderate correlation between ATAC-seq peak count and gene length is the most straightforward of the three results to interpret. While chromatin accessibility is determined by specific molecular recognition events: transcription factor binding, nucleosome remodeling, and the action of the transcriptional machinery, the number of functional regulatory elements that can reside within a locus is physically constrained by its genomic extent. Longer genes occupy more linear genomic space and contain more intronic sequence. Since a substantial fraction of active cis-regulatory elements, including tissue-specific enhancers and silencers, are located within intronic regions of the gene they regulate, longer genes accumulate more such elements by architectural necessity rather than by any non-specific scaling of accessibility with length (Shlyueva et al., 2014; Pennacchio et al., 2013). This is an important distinction: the correlation arises not because ATAC-seq peaks accumulate stochastically with length, but because the regulatory architecture of longer loci is inherently more complex, containing more distinct functional elements within their boundaries.

The near-absence of correlation between POP-seq peak count and both gene length and transcript abundance is considerably more striking and reflects a much more complex regulatory ecosystem. Here, the authors present three distinct but interacting mechanisms that could provide an explanation for the results and are likely amplified in the context of immortalized cell lines: RNA compartmentalization and sequestration, dynamic structural constraints on RBP accessibility, and RBP sequestration by competing transcripts.

Post-transcriptional regulation in eukaryotic cells is fundamentally compartmentalized. A transcript's measured abundance by RNA-seq reflects its total steady-state pool, but POP-seq captures only those copies that are physically accessible and occupied by RBPs at the time of the experiment. Critically, a substantial fraction of cytoplasmic mRNAs in cancer-derived and immortalized cell lines is sequestered into P-bodies and stress granules, and largely inaccessible to the broader RBP pool. Cancer cells, including leukemic cell lines such as K562, harbor aberrantly elevated numbers of P-bodies that selectively sequester specific mRNAs, potentially shielding them from detection by interaction-based assays. The consequence is that a highly abundant transcript that is predominantly sequestered into P-bodies may show far fewer POP-seq peaks than its steady-state FPKM value would predict, directly decoupling peak count from transcript abundance.

Furthermore, the accessibility of binding sites within a transcript to cognate RBPs is not determined solely by sequence composition but is critically regulated by RNA secondary and tertiary structure, which is itself dynamic and context-dependent. Structural accessibility of RBP binding motifs, the degree to which a sequence motif is single-stranded and spatially exposed, is a major determinant of in vivo RBP binding, and calculation of predicted structural accessibility substantially improves the accuracy of predicting in vivo binding sites beyond sequence motif alone. Importantly, the structures that constrain or expose binding sites in vivo are not those of the mature, fully folded transcript alone. In vivo probing of nascent RNAs has demonstrated that cotranscriptional folding is a kinetically driven process in which short-range secondary structures form immediately as the transcript is synthesized, while long-range interactions are transiently sequestered into non-native conformations until distal complementary sequences become available. This means that the structural landscape of a transcript, and therefore the accessibility of its RBP binding sites, is determined by the kinetics of its synthesis, processing, and folding pathway, not merely by its equilibrium thermodynamic structure or its abundance. Two transcripts of similar abundance can present radically different RBP-accessible surfaces depending on their cotranscriptional folding trajectories, the presence of RNA helicases, and the co-occupancy of other RBPs, all of which vary independently of transcript abundance or length.

Even if a transcript is cytoplasmic, accessible, and structurally permissive, its actual RBP occupancy depends on the global competitive landscape of all binding sites in the cell. The availability of RBPs is under the control of cell-intrinsic and extracellular cues, and the RBP components of assembled ribonucleoprotein complexes coordinately determine mRNA stability, localization, and translation. In immortalized and cancer-derived cell lines, this competitive landscape is substantially altered relative to normal primary cells: oncogenic transcripts are frequently overexpressed at very high abundance, and the RBP pool is dysregulated at the expression level. A small number of highly overexpressed oncogenic transcripts can effectively titrate particular RBPs away from their normal target repertoire, reducing the occupancy of other transcripts regardless of their abundance.

Taken together, these three mechanisms subcellular RNA compartmentalization and P-body sequestration, dynamic cotranscriptional structural constraints on binding site accessibility, and competitive RBP titration by overexpressed transcripts could provide a coherent and mutually reinforcing explanation for the observed decoupling of POP-seq peak count from both gene length and transcript abundance. Each mechanism is amplified in the context of immortalized and cancer-derived cell lines relative to what would be expected in healthy primary tissue, suggesting that the weak correlations observed here may themselves be a biological signature of the post-transcriptional dysregulation characteristic of these cellular contexts.

**Supplemental Figure 1.**

Scatter plots of gene wise correlations across 3 immortal human cell lines (HEK293, HepG2, and K562) RR category indicated by color (Predominantly Transctiptionally Regulated in green, Balanced Regulation in pink, and Predominantly Post-Transcriptionally Regulated in orange). **A.** Scatter plot depicting the gene-wise correlation between number of observed ATAC-seq peaks and gene length in HEK293 cells. **B.** Scatter plot depicting the gene-wise correlation between number of observed ATAC-seq peaks and gene length in HepG2 cells.**C.** Scatter plot depicting the gene-wise correlation between number of observed ATAC-seq peaks and gene length in K562 cells. **D.** Scatter plot depicting the gene-wise correlation between number of observed POP-seq peaks and gene length in HEK293 cells. **E.** Scatter plot depicting the gene-wise correlation between number of observed POP-seq peaks and gene length in HepG2 cells. **F.** Scatter plot depicting the gene-wise correlation between number of observed POP-seq peaks and gene length in K562 cells. **G.** Scatter plot depicting the gene-wise correlation between number of observed POP-seq peaks and relative gene frequency (FPKM) in HEK293 cells. **H.** Scatter plot depicting the gene-wise correlation between number of observed POP-seq peaks and relative gene frequency (FPKM) in HepG2 cells. **I**. Scatter plot depicting the gene-wise correlation between number of observed POP-seq peaks and relative gene frequency (FPKM) in HEK293 cells.

**Supplemental Table 1.**

| Gene Window  Upstream / Downstream (bp) | 0 / 0 | 1000 / 100 | 2500 / 250 | 5000 / 500 | 10000 / 1000 |
| --- | --- | --- | --- | --- | --- |
| Predominantly Transcriptionally Regulated (Hek293) | 5984  (38.6%) | 6456  (38.4%) | 6758  (39.5%) | 7138  (41.0%) | 7890  (44.4%) |
| Balanced (Hek293) | 1512  (9.75%) | 1644  (9.78%) | 1682  (9.84%) | 1718  (9.87%) | 1576  (8.87%) |
| Predominantly Post-Transcriptionally Regulated (Hek293) | 8007  (51.6%) | 8711  (51.8%) | 8650  (50.6%) | 8549  (49.1%) | 8301  (46.7%) |
| Predominantly Transcriptionally Regulated (HepG2) | 7143  (53.7%) | 7745  (54.4%) | 8029  (54.4%) | 8398  (57.3%) | 9064  (60.9%) |
| Balanced (HepG2) | 1136  (8.54%) | 1185  (8.33%) | 1215  (8.32%) | 1206  (8.23%) | 1173  (7.88%) |
| Predominantly Post-Transcriptionally Regulated (HepG2) | 5021  (37.8%) | 5297  (37.2%) | 5188  (37.2%) | 5044  (34.4%) | 4655  (31.3%) |
| Predominantly Transcriptionally Regulated (K562) | 6429  (55.15) | 7057  (56.0%) | 7338  (57.5%) | 7672  (59.3%) | 8320  (63.4%) |
| Balanced (K562) | 1102  (9.44%) | 1194  (9.47%) | 1182  (9.26%) | 1180  (9.13%) | 1047  (7.98%) |
| Predominantly Post-Transcriptionally Regulated (K562) | 4142  (35.5%) | 4350  (34.5%) | 4238  (33.2%) | 4077  (31.5%) | 3748  (28.6%) |

**Supplementary Table 2.**

| Gene Window  Upstream / Downstream (bp) | 0 / 0 | 1000 / 100 | 2500 / 250 | 5000 / 500 | 10000 / 1000 |
| --- | --- | --- | --- | --- | --- |
| Predominantly Transcriptionally Regulated (Hek293) | 0.87 | 0.9 | 0.95 | 1.00 | 1.00 |
| Balanced (Hek293) | 0.45 | 0.67 | 0.8 | 1.00 | 0.64 |
| Predominantly Post-Transcriptionally Regulated (Hek293) | 0.84 | 0.9 | 0.95 | 1.00 | 0.960 |
| Predominantly Transcriptionally Regulated (HepG2) | 0.85 | 0.92 | 0.96 | 1.00 | 1.00 |
| Balanced (HepG2) | 0.34 | 0.62 | 0.78 | 1.00 | 0.61 |
| Predominantly Post-Transcriptionally Regulated (HepG2) | 0.86 | 0.98 | 0.99 | 1.00 | 0.91 |
| Predominantly Transcriptionally Regulated (K562) | 0.84 | 0.920 | 0.96 | 1.00 | 1.00 |
| Balanced (K562) | 0.33 | 0.64 | 0.78 | 1.00 | 0.61 |
| Predominantly Post-Transcriptionally Regulated (K562) | 0.85 | 0.99 | 0.99 | 1.00 | 0.910 |
